# Adjustment disorder in cancer patients after treatment: prevalence and acceptance of psychological treatment

**DOI:** 10.1007/s00520-021-06530-0

**Published:** 2021-10-02

**Authors:** F. E. Van Beek, L. M. A. Wijnhoven, J. A. E. Custers, K. Holtmaat, B. H. De Rooij, N. J. E. Horevoorts, E. J. Aukema, S. Verheul, S. E. J. Eerenstein, L. Strobbe, I. M. Van Oort, M. R. Vergeer, J. B. Prins, I. M. Verdonck-de Leeuw, F. Jansen

**Affiliations:** 1 Department of Clinical, Neuro & Developmental Psychology, Amsterdam Public Health Research Institute, Vrije Universiteit Amsterdam, Amsterdam, The Netherlands; 2 Department of Medical Psychology, Radboud Institute of Health Sciences, Radboudumc Nijmegen, Nijmegen, The Netherlands; 3 Department of Research, Netherlands Comprehensive Cancer Organisation (IKNL), Utrecht, The Netherlands; 4 CoRPS - Center of Research On Psychology in Somatic Diseases, Department of Medical and Clinical Psychology, Tilburg University, Tilburg, The Netherlands; 5 Ingeborg Douwes Centrum, Center for Psycho-Oncology, Amsterdam, The Netherlands; 6 Department of Medical Psychology, CWZ Nijmegen, Nijmegen, The Netherlands; 7 Department of Otolaryngology-Head and Neck Surgery, Cancer Center Amsterdam, Amsterdam UMC, VUmc, PO Box 7057, 1007 MB Amsterdam, The Netherlands; 8 Department of Oncological Surgery, CWZ Nijmegen, Amsterdam, The Netherlands; 9 Department Urology, Radboudumc Nijmegen, Nijmegen, The Netherlands; 10 Department of Radiation Oncology, Cancer Center Amsterdam, Amsterdam UMC, VUmc, Amsterdam, The Netherlands

**Keywords:** Psychology, Cancer, Adjustment disorder, Prevalence, Psychological treatment, Acceptance of psychological treatment

## Abstract

**Purpose:**

To investigate the prevalence of adjustment disorder (AD) among cancer patients and the acceptance of psychological treatment, in relation to sociodemographic, clinical, and psychological factors.

**Methods:**

Breast, prostate, and head and neck cancer patients of all stages and treatment modalities (*N* = 200) participated in this observational study. Patients completed the Hospital Anxiety and Depression Scale, Checklist Individual Strength, Distress Thermometer and problem list. Patients with increased risk on AD based on these questionnaires were scheduled for a diagnostic interview. Patients diagnosed with AD were invited to participate in a randomized controlled trial on the cost-effectiveness of psychological treatment. Participation in this trial was used as a proxy of acceptance of psychological treatment. Logistic regression analyses were used to investigate associated factors.

**Results:**

The overall prevalence of AD was estimated at 13.1%. Sensitivity analyses showed prevalence rates of AD of 11.5%, 15.0%, and 23.5%. Acceptance of psychological treatment was estimated at 65%. AD was associated both with being employed (OR = 3.3, CI = 1.3–8.4) and having a shorter time since diagnosis (OR = 0.3, CI = 0.1–0.8).

**Conclusion:**

Taking sensitivity analysis into account, the prevalence of AD among cancer patients is estimated at 13 to 15%, and is related to being employed and having a shorter time since diagnosis. The majority of cancer patients with AD accept psychological treatment.

## Background

Cancer patients may experience psychological problems [1]. One of these psychological problems is adjustment disorder (AD). According to the Diagnostic and Statistical Manual of Mental Disorders (DSM-V)[2], AD occurs when adaptation to a significant identifiable life stressor, such as cancer, fails.

In a meta-analysis of Mitchell et al. (2011) [3], the prevalence of AD among cancer patients was estimated at 19.4% (confidence interval (CI) 14.5–24.8%). More recent studies showed prevalence rates ranging from 6 to 17% [4–7]. This variability in prevalence rates may result from methodological differences among studies, as well as from different diagnostic procedures for AD. In the Netherlands, a national guideline on AD has been available since 2016, which includes an assessment procedure for AD diagnosis [8]. Another reason for the observed variation may be that prevalence rates differ among cancer groups. A study of Mehnert et al. [4] showed that the prevalence rate of AD varied between tumor types, with the lowest rate of 2.9% in rectal cancer patients and the highest rate of 16.5% in head and neck cancer patients. Other studies demonstrated that patients who were female, more highly educated, diagnosed with a more advanced tumor stage, and living in rural areas, and who lacked physical exercise were more frequently diagnosed with AD [5, 9].

Concerning the usage of psychological treatment, a previous meta-analysis of Brebach et al. [10] showed that 60% of cancer patients exhibiting distress wanted psychological treatment. A higher usage of psychological treatments was associated with a more recent cancer diagnosis, remote compared to face-to-face treatment and psychological treatment provided by a nurse compared to other psychosocial professionals [9]. Other studies showed that patients who were younger, female, and more highly educated were more likely to accept psychological treatment [11–13]. However, no study so far has focused on the acceptance of psychological treatment for AD in cancer patients.

In summary, there is inconclusive or limited evidence of the prevalence of AD and the acceptance of psychological treatment for AD among cancer patients, as well as its associated factors. The aim of this study was to investigate (1) the prevalence of AD among cancer patients in relation to sociodemographic and clinical factors; (2) to investigate sociodemographic, clinical, and psychological factors associated with AD among cancer patients with an increased risk for AD; and (3) to investigate the acceptance of psychological treatment among patients with AD in relation to sociodemographic, clinical, and psychological factors. Factors associated with AD among cancer patients in general and cancer patients with an increased risk for AD were investigated separately, as patient-reported outcome measures (PROMS) are increasingly used in clinical practice to identify patients with psychological problems. Due to the design of this study, the association between psychological factors and prevalence of AD could only be investigated among patients with an increased risk for AD.

## Methods

### Design, participants, and study procedures

This observational study recruited cancer patients from Amsterdam UMC, Canisius Wilhelmina Hospital and Radboudumc, the Netherlands, between September 2019 and January 2020. The study was part of a randomized controlled trial (RCT) on the effectiveness and cost-utility of tailored psychological treatment targeting cancer patients with AD [14]. Patients were included, when they (1) were diagnosed with cancer (all types and stages, except non-melanoma skin cancer) between July 2004 and July 2019, (2) were aged ≥18 years, and (3) completed primary cancer treatment with curative or palliative intent (all treatment modalities, except for endocrine therapy in breast and prostate cancer).

Random selections of patients were drawn by the Netherlands Cancer Registry (NCR) which registers all newly diagnosed cancer patients. Recruitment started among breast, prostate, and head and neck cancer patients. Due to the COVID-19 pandemic, patients with other cancer diagnoses could not be recruited. The (former) treating physician checked the eligibility of the patients. After confirming eligibility, a patient information letter with informed consent form was sent to the patient by mail. After consenting, the patient was asked to complete the study questionnaire measuring their risk for AD.

Study procedures were approved by the Medical Ethical Committee of VUmc and followed the Dutch Medical Research Involving Human Subjects Act.

### Primary outcome

The primary outcomes were prevalence of AD and acceptance of psychological treatment. Prevalence was measured through a two-phase approach including a screening procedure and a diagnostic interview.

Patients were screened on their risk for AD using the Hospital Anxiety and Depression Scale (HADS), Distress Thermometer (DT), and problem list. The HADS is a psychometrically validated 14‐item self-report questionnaire that measures symptoms of anxiety (HADS-A) and depression (HADS-D) in the last week. Also, a total HADS (HADS-T) score can be calculated ranging from 0 (no distress) to 42 (severe distress) [15]. The DT measures the level of distress experienced in the last week on a scale ranging from 0 (no distress) to 10 (extreme distress) [16]. The problem list measures 47 different problems in the last week, including an item on willingness to talk to an expert, followed by a question on type of expert (psychologist, social worker, dietician, physiotherapist, nurse, peers or other) [16]. Increased risk for AD was defined as HADS-total ≥11 or DT≥4 or willingness to talk with a psychologist or social worker [14].

Patients with an increased risk for AD were invited for a diagnostic interview either by telephone or face-to-face. The interviews were carried out by trained psychologists, who were registered in the expert database of the Dutch Association for Psycho-oncology (NVPO) or under supervision of a registered psychologist. All psychologists followed an E-Learning program on diagnosis and treatment of AD, which included a reader, videos, and an online assessment [8, 17]. The E-learning comprised several learning objectives including the definition of AD among cancer patients and how to describe symptoms along the criteria of the DSM-V. The psychologists completed a form per patient on DSM-V classification of AD (yes/no).

Patients diagnosed with AD were invited by the psychologist to participate in an RCT in which patients received tailored psychological treatment immediately or after a period of 6 months [14]. If a patient was interested in the RCT, a researcher gave further information via telephone and an information letter was sent. In the case that a patient did not respond, they were reminded after 1 week by telephone. Reasons not to participate were reported.

### Factors associated with AD and acceptance of psychological treatment

To investigate factors associated with AD and acceptance of psychological treatment, the HADS, DT and problem list, the Checklist Individual Strength (CIS) and questions on sociodemographic and clinical characteristics were used. HADS, DT, and problem list are described above. The CIS is a valid and reliable 20-item instrument to measure fatigue, concentration, motivation, and physical activity [18, 19]. A higher score (20–140) indicates a higher level of fatigue.

The socio-demographic questions focused on sex (male/female), age (years), marital status (yes/no), education level (high/low), and employment status (yes/no). Clinical data (tumor stage (I–II/III–IIII), treatment (single/multiple treatment), and time since diagnosis (less/more than 5 years after diagnosis)) and social economic status (high/middle/low) were obtained from the NCR.

### Statistical methods

Quantitative analyses were performed using the IBM Statistical package for the Social Science version 26. Descriptive statistics were generated for all baseline characteristics and outcome measures. To investigate selective non-response in phase 1 (screening), respondents and non-respondents were compared using independent *T*-test and chi-square test. In phase 2 (diagnostic interview), participants (those who completed the interview) and drop-outs (those with an increased risk but who did not complete the interview) were also compared. A *p*-value<0.05 was considered statistically significant.

To estimate the prevalence of AD among patients, the number of patients diagnosed with AD was divided by the total number of participants that completed the screening survey minus the total number of drop-outs in phase 2. In addition, sensitivity analyses were performed in which drop-outs of phase 2 were (a) all expected to have AD, (b) partly expected to have AD (the same prevalence as other patients in phase 2), and (c) all expected to have no AD. To estimate usage of psychological treatment, the number of patients who agreed to participate in the RCT was divided by the total number of patients diagnosed with AD.

Possible factors associated with (1) the prevalence of AD among all patients and (2) the prevalence of AD among patients with increased risk and (3) the acceptance of a psychological treatment were investigated using forward logistic regression analyses. Variables were entered one-by-one into the logistic regression model using a *p*-value<0.05. Since the HADS, DT, and problem list were used to identify patients with an increased risk for AD, these variables were not entered in the logistic regression models on the prevalence of AD among all cancer patients.

## Results

### Participants

**Fig. 1:**
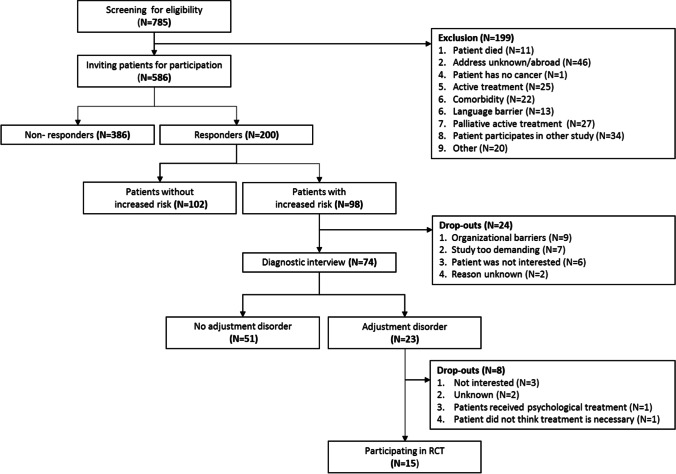
Flow diagram

Figure 1 shows the study flow diagram. Of the 785 cancer patients who were screened for eligibility, 586 patients were invited to participate in the study. There were significant differences between the patients who responded (*N*=200, 34%) and those who did not respond (*N*=386, 66%). Patients who responded were more often male, had a higher social economic status, and were more frequently diagnosed with prostate cancer and more often diagnosed with tumor stage I or II compared to patients who did not respond (Table 1). Characteristics of the study population (*N*=200) are shown in Table 2.

**Table 1:** Characteristics of responders and non-responders

Characteristics	Non-responders Part 1 (*N* = 386)	Responders Part 1 (*N* = 200)	*p*-value
Age mean (SD)	68 (10)	68 (10)	0.80
Gender			** < 0.001**
Male	109 (28%)	87 (44%)	
Female	277 (72%)	113 (57%)	
Social economic status			
Low	113 (29%)	39 (20%)	**0.002**
Middle	170 (44%)	81 ((41%)	
High	103 (27%)	80 (40%)	
Tumorsite			
Prostate	49 (13%)	56 (28%)	** < 0.001**
Breast	246 (64%)	98 (49%)	
Head and neck	91 (24%)	46 (23%)	
Tumor stage			
I–II	316 (82%)	151 (76%)	**0.001**
III–IV	69 (18%)	49 (25%)	
Time since diagnosis (years)			0.71
0–5	112 (29%)	53 (27%)	
> 5	274 (71%)	146 (73%)	

Abbreviation: *SD*, standard deviation

Result printed in bold is significant (*P*<0.05)

**Table 2:** Characteristics study population

	Responders (*N* = 200)	Patients without AD (*N* = 153)	Patients with Increased risk and no AD (*N* = 51)	Patients with AD (*N* = 23)	Patients with treatment (*N* = 15)	Patient without treatment (*N* = 8)
Age mean (SD)	68 (10)	69 (9)	68 (9)	63 (13)	62 (13)	63 (12)
Gender						
Female	113 (57%)	81 (53%)	27 (53%)	14 (61%)	8 (53%)	6 (75%)
Married (yes/no)						
Yes	136 (68%)	108 (71%)	33 (65%)	13 (57%)	8 (53%)	5 (63%)
Employment status (yes/no)						
Yes	49 (25%)	34 (22%)	8 (16%)	12 (52%)	8 (53%)	3 (38%)
Education (high/low)						
High	115 (58%)	88 (58%)	29 (57%)	12 (52%)	7 (47%)	5 (63%)
Tumor site						
Prostate	56 (28%)	46 (30%)	10 (20%)	4 (18%)	4 (27%)	2 (25%)
Breast	98 (49%)	71 (46%)	26 (51%)	13 (57%)	7 (47%)	6 (75%)
Head and neck	46 (23%)	36 (24%)	15 (29%)	6 (26%)	4 (27%)	0
Tumor stage (I–II/III–IV))						
III–IV	49 (25%)	38 (25%)	25 (49%)	4 (17%)	3 (20%)	1 (13%)
Treatment^1^						
Single treatment	102 (51%)	78 (51%)	26 (51%)	13 (57%)	10 (67%)	3 (38%)
Surgery	76 (38%)	59 (39%))	19 (37%)	10 (44%)	8 (53%)	2 (25%)
Radiotherapy	25 (13%)	18 (12%)	7 (14%)	3 (13%)	2 (13%)	0
Chemotherapy	1 (1%)	1 (1%)	0	0	0	0
Multiple treatment	96 (48%)	73 (48%)	25 (49%)	10 (44%)	5 (33%)	5 (63%)
Surgery + radiotherapy	41 (21%)	35 (23%)	12 (24%)	0	0	0
Surgery + chemotherapy	16 (8%)	10 (7%)	1 (2%)	5 (22%)	1 (7%)	4 (50%)
Radiotherapy + chemotherapy	12 (6%)	9 (6%)	3 (6%)	1 (4%)	1 (7%)	0
Surgery + radiotherapy + chemotherapy	27 (14%)	19 (12%)	9 (18%)	4 (17%)	3 (20%)	1 (13%)
Hormone therapy	60 (30%)	44 (29%)	17 (33%)	12 (52%)	5 (33%)	5 (63%)
Time since diagnosis (years)						
> 5	145 (73%)	119 (78%)	40 (78%)	12 (52%)	6 (40%)	6 (75%)
Psychological outcome scores mean (SD)						
HADS-T^**1**^	7.4 (6.9)	5.5 (5.7)	10.8 (6.4)	13.9 (6.9)	14.3 (6.6)	13.3 (7.7)
HADS-A^**1**^	4.1 (3.9)	3.1 (3.3)	7.9 (4.3)	7.9 (4.3)	8.6 (4.5)	6.5 (3.9)
HADS-D^**1**^	3.3 (3.6)	2.5 (3.0)	2.1 (3.9)	6.1 (3.9))	5.7 (3.5)	6.8 (4.6)
DT^1^	3.6 (2.8)	2.7 (2.6)	5.7 (6.0)	6.5 (1.9)	6.6 (1.5)	6.4 (2.4)
CIS^1^	58.8 (29.4)	54.6 (25.9)	75.2 (24.4)	81.7 (27.8)	83.6 (26.0)	77.8 (33.0)
Items on problem list (yes)						
Practical problems	71 (36%)	44 (29%)	29 (57%)	13 (57%)	9 (60%)	4 (50%)
Family and social	30 (15%)	17 (11%)	10 (20%)	8 (35%)	5 (33%)	3 (38%)
Emotional	111 (56%)	81 (52%)	41 (80%)	19 (83%)	14 (93%)	5 (63%)
Religious or spiritual	39 (20%)	23 (15%)	12 (24%)	8 (35%)	5 (33%)	3 (38%)
Physical	163 (82%)	117 (77%)	50 (98%)	22 (96%)	14 (93%)	8 (100%)
Willingness to talk to an expert^1^						
Yes/maybe	66 (33%)	39 (26%)	25 (49%)	21 (91%)	14 (93%)	7 (88%)

Abbreviations: *AD*, adjustment disorder; *CIS*, Checklist Individual Strength; *DT*, distress thermometer; *HADS*, Hospital Anxiety and Depression Scale; *-A*, anxiety subscale; *-D*, depression subscale; *-T*, total score

^1^Missing data: treatment (2), HADS-T (2), HADS-A (1), HADS-D (1), DT (1), CIS (6), willingness to talk (1)

### Prevalence of AD

Of all 200 patients that completed the survey, 98 patients had an increased risk for AD (49%) and were invited for a diagnostic interview (Figure 1). Of these 98 patients with an increased risk, 74 patients agreed to participate in a diagnostic interview (participation rate 75%). There were no significant differences between participants and drop-outs except that patients who dropped out reported more frequently that they were not willing to talk to an expert (Table 3).

**Table 3:** Characteristics of patients with an increased risk for AD who did and did not participate in the diagnostic interview

Characteristics	Patients with an increased risk who had an interview in part 2 (*N* = 74)	Drop-outs part 2 (*N* = 24)	*p*-value
Age mean (SD)	66 (11)	67 (11)	0.63
Gender			0.09
Female	41 (55%)	18 (75%)	
Married (yes/no)			0.98
Yes	28 (38%)	15 (63%)	
Employed (yes/no)			0.37
Yes	19 (26%)	4 (17%)	
Education (high/low)			0.54
High	41 (55%)	15 (63%)	
Tumor site			0.85
Prostate	16 (22%)	4 (17%)	
Breast	39 (53%)	14 (58%)	
Head and neck	19 (26%)	6 (25%)	
Tumor stage (I–II/III–IV)			0.64
III–IV	16 (22%)	13 (54%)	
Treatment			0.56
Single treatment	39 (53%)	11 (46%)	
Surgery	29 (39%)	7 (29%)	
Radiotherapy	10 (14%)	4 (17%)	
Chemotherapy	0	0	
Multiple treatment	35 (47%)	13 (54%)	
Surgery + radiotherapy	12 (16%)	6 (25%)	
Surgery + chemotherapy	6 (8%)	1 (4%)	
Radiotherapy + chemotherapy	4 (5%)	2 (8%)	
Surgery + radiotherapy + chemotherapy	13 (18%)	4 (17%)	
Hormone therapy	27 (37%)	6 (25%)	0.30
Time since diagnosis (years)			0.74
> 5	52 (70%)	16 (67%)	
Psychological outcome mean (SD)			
HADS-T	11.9 (3.7)	13.0 (7.3)	
HADS-A	6.5 (4.0)	7.0 (3.8)	0.59
HADS-D	5.4 (3.6)	6.0 (4.4)	0.50
DT	5.9 (6.0)	6.1 (1.4)	0.65
CIS	77.6 (25.4)	84.0 (28.2)	0.27
Items on problem list (yes)			
Practical problems	42 (57%)	14 (58%)	0.89
Family and social	18 (24%)	5 (21%)	0.73
Emotional	60 (81%)	20 (83%)	0.80
Religious or spiritual	20 (27%)	8 (33%)	0.55
Physical	72 (97%)	24 (100%)	0.41
Willingness to talk to an expert			
Yes/maybe	46 (62%)	6 (25%)	**0.002**

Abbreviations: *AD*, adjustment disorder; *CIS*, Checklist Individual Strength; *DT*, distress thermometer; *HADS*, Hospital Anxiety and Depression Scale; *-A*, anxiety subscale; *-D*, depression subscale; *-T*, total score; *SD*, standard deviation

Result printed in bold is significant (*P*<0.05)

Of the 74 participants with an increased risk for AD and who participated in a diagnostic interview, 23 patients were diagnosed with AD (31%). The overall prevalence rate of AD was estimated at 13.1%. Sensitivity analyses in which the 24 patients who dropped out were all expected to have AD, partly expected to have AD, or all expected to have no AD, showed prevalence rates of 23.5%, 15.0%, and 11.5% respectively. Multivariate analysis showed that overall AD was significantly associated with employment status and time since diagnosis (Table 4). The prevalence of AD was higher in patients who were employed (odds ratio (OR)=3.3, 95%CI=1.3–8.4) and higher in patients diagnosed less than 5 years ago (OR=0.3, 95%CI=0.1–0.8). Among patients who participated in the diagnostic interview (*N*=74), AD was significantly associated with employment status, time since diagnosis, and willingness to talk to an expert (Table 4). The prevalence of AD was higher in patients who were employed (OR=3.2, 95%CI=1.3–8.4), patients who were diagnosed less than 5 years prior to the study (OR=0.3, 95%CI=0.007–0.9), and patients who were willing to talk to a psychologist or social worker (OR=9.2, 95%CI=1.9–45.6).

### Acceptance of psychological treatment

Of all 23 patients diagnosed with AD, 15 patients participated in the RCT (65%) (Figure 1). Univariate analysis showed that acceptance of treatment was not significantly associated with any of the investigated factors (Table 4).

**Table 4:** Variables associated with AD and acceptance of psychological treatment

Variables	Presence of AD among all patients (*N* = 176)		Presence of AD among patients with increased risk (*N* = 74)		Acceptance of psychological treatment among patients with AD (*N* = 23)
	Univariate OR [95%CI]	Multivariate OR [95%CI]	Univariate OR [95%CI]	Multivariate OR [95%CI]	Univariate OR [95%CI]
Clinical and demographic					
Mean age	0.9 [0.9–1.0]		1.0 [0.9–1.0]		1.0 [0.9–1.1]
Gender (reference = male)	1.4 [0.6–3.9]		1.4 [0.5–3.8]		0.4 [0.1–2.5]
Marital status (reference = no marital status)	0.5 [0.2–1.3]		0.7 [0.3–1.9]		0.7 [0.1–4.0]
Employment status (references = no employment status)	3.2 [1.3–7.9]**	3.4 [1.3–8.5]**	4.9 [1.6–15.0]*	4.4 [1.2–16.01]*	1.9 [0.3–11.0]
Education (reference = lower)	0.8 [0.3–1.9]		0.8 [0.3–2.2]		0.5 [0.1–3.0]
Tumor site (reference = prostate)					
Breast	1.4 [0.5–4.0]		0.8 [< 0.01–2.0]		N/A^1^
Head and neck	0.9 [0.2–3.3]		0.4 [ 0.2–2.8]		N/A^1^
Tumor stage (reference = I–II)	0.6 [0.2–1.9]		0.6 [0.2–1.9]		1.8 [0.2–20.2]
Treatment (reference = single)	0.8 [0.3–2.0]		0.8 [0.3–2.2]		0.3 [0.1–1.8]
Years since diagnosis (reference = 0–5)	0.3 [0.1–0.8]**	0.3 [0.1–0.8]**	0.3 [0.1–0.9]*	0.3 [0.07–0.9]*	0.2 [< 0.1–1.5]
Psychological outcomes					
HADS-T			1.1 [1.0–1.2]		1.0 [ 0.9–1.2]
HADS-A			1.1 [1.0–1.3]		1.1 [0.9–1.4]
HADS-D			1.1 [0.9–1.2]		0.9 [0.7–1.2]
DT			1.3 [1.0–1.6]		1.1 [0.7–1.7]
CIS			1.0 [1.0–1.0]		1.0 [1.0–1.0]
Items on problem list (reference = no)					
Practical			1.0 [0.4–2.7]		1.5 [0.3–8.4]
Family and social			2.2 [0.7–6.9]		0.8 [0.1–5.0]
Emotional			1.2 [0.3–4.2]		8.4 [ 0.7–100.6]
Religious or spiritual			1.7 [0.6–5.1]		0.8 [0.1–5.0]
Physical			0.4 [0.03–7.3]		N/A^1^
Willingness to talk to an expert			10.9 [2.3–51.5]*	9.2 [1.9–45.6]*	2.0 [0.1–37.0]

Abbreviations: *AD*, adjustment disorder; *CIS*, Checklist Individual Strength; *DT*, distress thermometer; *HADS*, Hospital Anxiety and Depression Scale; *-A*, anxiety subscale; *-D*, depression subscale; *-T*, total score; *OR*, odds ratio; *CI*, confidence interval

^1^Analyses reported with “N/A” were not applicable due to limited sample size

^*^p-value < 0.05, **p-value < 0.01

Results printed in bold are significant (*P*<0.05)

## Discussion

This study investigated the prevalence of AD among cancer patients and the acceptance of psychological treatment for AD, in relation to sociodemographic, clinical, and psychological factors. Overall prevalence rate of AD was estimated at 13%. Being employed and being diagnosed less than 5 years prior to the study were significantly associated with AD. It was estimated that 65% of patients with AD were willing to accept psychological treatment. None of the investigated factors was associated with acceptance of psychological treatment.

The prevalence rate of AD should be viewed within the light of the sensitivity analyses in which prevalence rates of 24%, 15%, and 12% were found. As there were no significant differences in sociodemographic, clinical, and psychological characteristics, except from willingness to talk to an expert, between patients with an increased risk for AD who did and did not participate in the diagnostic interview, we assume that scenario b (i.e., prevalence of AD is the same among patients with an increased risk for AD who did and did not participate in the diagnostic interview) is most acceptable. Therefore, a prevalence rate of 13–15% is expected to be most plausible. The prevalence rate of 13–15% is in line with two previous studies reporting prevalence rates of 12% [4, 5]. A previous meta-analysis showed a higher prevalence rate of 19.4% [3], and another recent study showed a prevalence rate of 17% [7]. The studies with similar prevalence rates used a comparable two-step method for diagnosing AD as performed in this study, albeit that they used a different screening instrument (PHQ-9) [4, 5]. Such a two-step approach has been proven to be valid and efficient [20] and is in accordance with the Dutch guideline on AD [8]. A drawback of this procedure is that patients may have been missed who had a low score on the screening questionnaires who should be diagnosed with AD. This may explain the somewhat higher prevalence rates of 17% [7] and 19% [3] in studies in which all patients received a diagnostic interview. Another explanation may be the absence of clear criteria to diagnose AD, as strict diagnostic criteria for AD in the DSM-V are lacking [21]. As a consequence, the diagnosis of AD may be prone to a psychologist’s individual interpretation of the criteria.

The current study demonstrated that being employed, being diagnosed less than 5 years prior to the study, and being willing to talk to an expert are associated with AD, while sociodemographic factors as age, sex, education, and marital status, and clinical factors as cancer type, stage, and treatment were not. This is in contrast to previous studies reporting that being female, younger, unmarried, more highly educated, and diagnosed with a more advanced tumor stage are associated with AD [5, 9]. An explanation might be the relatively small sample size of our study that may have failed to detect smaller differences. Also, in our study we included breast cancer, head and neck cancer and prostate cancer patients, whereas previous studies focused on breast cancer patients only or a combination of 13 different tumor types [5, 9]. The distribution of sociodemographic and clinical characteristics such as gender, education level, and tumor stage may consequently differ among studies. Another explanation may be that in contrast to our study, in previous studies time since diagnosis and employment status were not investigated while these factors might be more important than other factors.

Cancer patients who have to manage multiple tasks (e.g., work, housekeeping, children) may perceive cancer-related stressors as a higher burden compared to those with less tasks (e.g., those who are not employed) and therefore may be more vulnerable for developing distress [22, 23] or psychiatric disorders as AD. Although the association between paid work and AD has not been reported or studied in previous research, it is largely in line with previous research that showed an association between work and psychological symptoms [24, 25]. The same holds for the association between willingness to talk to a psychologist or social worker, which has previously been demonstrated to be associated with higher psychological distress [16, 26]. The finding that shorter time since diagnosis is associated with AD confirms previous reviews showing that psychiatric disorders as well as psychological symptoms are highest at time since diagnosis and slightly decrease over time [3, 27]. However, there are no longitudinal studies investigating AD over time, so further research is needed to investigate whether AD decreases, increases, or fluctuates over time. Longitudinal research may also clarify whether AD should be regarded as a transient diagnosis or as a disorder that should be treated to prevent a shift to another type of diagnosis (e.g., depression disorder) [28, 29].

Of the 23 patients diagnosed with AD in our study, 65% were willing to participate in an RCT on the effectiveness and cost-utility of psychological treatment for AD, and accepted psychological treatment. This is in line with the results of the meta-analysis of Brebach et al. [10] who found a pooled usage rate of 60% for psychological treatment among cancer patients. Brebach et al. [10] suggested that the possibility of assignment to a non-intervention group, and interventions delivered by telephone compared to face-to-face increased the usage of psychological interventions. A recent qualitative study showed that, from the patient’s perspective, the organization of psychological treatment targeting cancer patients should focus on easy accessibility and availability, delivery by specialized psychologists, and integration in medical cancer care. Online and group therapy are acceptable, but individual face-to-face therapy is preferred [30]. We did not find factors associated with the acceptance of psychological treatment in the current study, which is possibly due to the limited statistical power. Further quantitative research is needed to investigate factors associated with the acceptance of psychological treatment for AD [10–12].

### Study limitations

A strength of our study is the two-step approach to diagnose AD. A limitation is that, due to the COVID-19 pandemic, we had to stop recruiting patients earlier than planned, which resulted in 200 patients with breast, prostate, and head and neck cancer instead of the planned 3000 patients with various types of cancer [14]. The low response rate of 34%, and significant differences between the responders and non-responders might also limit the representativeness of this study. Another limitation is that the included patients were comparatively older and time since diagnosis was relatively longer. Finally, the results of this study are applicable to the situation before the COVID-19 pandemic. The prevalence of AD and acceptance of psychological treatment might be different during or after this pandemic. Nevertheless, the findings in this study can serve as benchmark for future studies investigating AD and the acceptance of psychological treatment among cancer patients.

### Clinical implications

As the prevalence of AD is substantial and acceptance of psychological treatment is high, implementation of screening procedures to identify patients with AD in routine care is recommended. However, effectiveness and cost-effectiveness of psychological treatment of AD remain to be answered. An ongoing RCT will provide more evidence [14]. Further research should also focus on barriers to accept psychological treatment among cancer patients with AD as there is still a large gap between patients who may need treatment and patients who actually accept and use psychological treatment.

## Conclusion

The prevalence of AD among cancer patients is estimated at 13 to 15%. AD among all cancer patients was found to be significantly associated with being employed and shorter time since diagnosis. AD among cancer patients who participated in the diagnostic interview was found to be significantly associated with being employed, shorter time since diagnosis and willingness to talk to an expert. The majority of cancer patients with AD accept psychological treatment.

## Data Availability

The datasets generated during and analyzed during the current study are available from the corresponding author on reasonable request.

## Abbreviations

AD: Adjustment disorder
CIS: Checklist Individual Strength
DT: Distress thermometer
NVPO: Dutch Association for Psycho-oncology
HADS: Hospital Anxiety and Depression Scale
NCR: Netherlands Cancer Registry
PROMS: Patient-reported outcome measures

## References

[1] Stein KD, Syrjala KL, Andrykowski MA (2008). Physical and psychological long-term and late effects of cancer. Cancer.

[2] Nuckols CC, Nuckols CC (2013). The diagnostic and statistical manual of mental disorders, (DSM-5).

[3] Mitchell AJ, Chan M, Bhatti H, Halton M, Grassi L, Johansen C (2011). Prevalence of depression, anxiety, and adjustment disorder in oncological, haematological, and palliative-care settings: a meta-analysis of 94 interview-based studies. Lancet Oncol.

[4] Mehnert A, Brähler E, Faller H, Härter M, Keller M, Schulz H (2014). Four-week prevalence of mental disorders in patients with cancer across major tumor entities. J Clin Oncol.

[5] Hund B, Reuter K, Härter M, Brähler E, Faller H, Keller M (2016). Stressors, symptom profile, and predictors of adjustment disorder in cancer patients. Results from an epidemiological study with the Composite International Diagnostic Interview, adaptation for oncology (CIDI-O). Depress Anxiety.

[6] Blázquez MH, Cruzado JA (2016). A longitudinal study on anxiety, depressive and adjustment disorder, suicide ideation and symptoms of emotional distress in patients with cancer undergoing radiotherapy. J Psychosom Res.

[7] Singer S, Meyer A, Wienholz S, Briest S, Brown A, Dietz A (2014). Early retirement in cancer patients with or without comorbid mental health conditions: a prospective cohort study. Cancer.

[8] Trimbos (2016) [Guidelines adjustment disorder in cancer patients]. Available via trimbos.nl. Accessed 21 march 2021

[9] Tang H-Y, Xiong H-H, Deng L-C, Fang Y-X, Zhang J, Meng H (2020). Adjustment disorder in female breast cancer patients: prevalence and its accessory symptoms. Future.

[10] Brebach R, Sharpe L, Costa DS, Rhodes P, Butow P (2016). Psychological intervention targeting distress for cancer patients: a meta-analytic study investigating uptake and adherence. Psychooncology.

[11] Faller H, Weis J, Koch U, Brähler E, Härter M, Keller M (2017). Utilization of professional psychological care in a large German sample of cancer patients. Psychooncology.

[12] Salmon P, Clark L, McGrath E, Fisher P (2015). Screening for psychological distress in cancer: renewing the research agenda. Psychooncology.

[13] Carlson LE, Waller A, Mitchell AJ (2012). Screening for distress and unmet needs in patients with cancer: review and recommendations. J Clin Oncol.

[14] Van Beek FE, Wijnhoven LM, Jansen F, Custers JA, Aukema EJ, Coupé VM (2019). Prevalence of adjustment disorder among cancer patients, and the reach, effectiveness, cost-utility and budget impact of tailored psychological treatment: study protocol of a randomized controlled trial. BMC psychology.

[15] Spinhoven P, Ormel J, Sloekers P, Kempen G, Speckens A, Van Hemert A (1997). A validation study of the Hospital Anxiety and Depression Scale (HADS) in different groups of Dutch subjects. Psychol Med.

[16] Tuinman MA, Gazendam-Donofrio SM, Hoekstra-Weebers JE (2008). Screening and referral for psychosocial distress in oncologic practice: use of the Distress Thermometer. Cancer.

[17] Workgroup adjustment disorders (2017) [Indication Adjustment disorder in cancer patients]. The Netherlands Organisation for Health Research and Development (ZonMw). Richtlijn - Aanpassingsstoornis bij patiënten met kanker (zonmw.nl)

[18] Worm-Smeitink M, Gielissen M, Bloot L, Van Laarhoven H, Van Engelen B, Van Riel P (2017). The assessment of fatigue: Psychometric qualities and norms for the Checklist individual strength. J Psychosom Res.

[19] Vercoulen J, Alberts M, Bleijenberg G (1999). Checklist Individual Strength (CIS). Gedragstherapie.

[20] Jacobi F, Wittchen H-U, Hölting C, Höfler M, Pfister H, Müller N (2004). Prevalence, co-morbidity and correlates of mental disorders in the general population: results from the German Health Interview and Examination Survey (GHS). Psychol Med.

[21] Baumeister H, Maercker A, Casey P (2009). Adjustment disorder with depressed mood. Psychopathology.

[22] Erikson EH (1994) Identity and the life cycle: WW Norton & Company

[23] Slater CL (2003). Generativity versus stagnation: an elaboration of Erikson’s adult stage of human development. J Adult Dev.

[24] Semple CJ, McCance T (2010). Parents’ experience of cancer who have young children: a literature review. Cancer Nurs.

[25] Mehnert A (2011). Employment and work-related issues in cancer survivors. Crit Rev Oncol Hematol.

[26] Baker-Glenn EA, Park B, Granger L, Symonds P (2011). Mitchell AJ (2011) Desire for psychological support in cancer patients with depression or distress: validation of a simple help question. Psychooncology.

[27] Krebber A, Buffart L, Kleijn G, Riepma I, De Bree R, Leemans C (2014). (2014) Prevalence of depression in cancer patients: a meta-analysis of diagnostic interviews and self-report instruments. Psychooncology.

[28] Takei N, Sugihara G (2006). Diagnostic ambiguity of subthreshold depression: minor depression vs adjustment disorder with depressive mood. Acta Psychiatrica Scandinavica..

[29] Kendler KS, Karkowski LM, Prescott CA (1999). Causal relationship between stressful life events and the onset of major depression. Am J Psychiatry.

[30] Schuit AS, Holtmaat K, Van Zwieten V, Aukema EJ, Gransier L, Cuijpers P (2021). Organizing psycho-oncological care for cancer patients: the patient’s perspective. Front Psychol.

